# The possible mechanisms of CYP2E1 interactions with HSP90 and the influence of ethanol on them

**DOI:** 10.1186/1472-6807-12-33

**Published:** 2012-12-17

**Authors:** Volodymyr O Kitam, Oksana V Maksymchuk, Mykola O Chashchyn

**Affiliations:** 1 Institute of molecular biology and genetics of NAS of Ukraine, Kyiv, Ukraine

## Abstract

**Background:**

Microsomal CYP2E1 metabolizes about 160 hydrophobic exogens, many of which are environmental pollutants. While metabolising xenobiotics CYP2E1 on one hand facilitates in their excretion and on the other hand activates them into the cytotoxins, which may damage the cell. Thus the CYP2E1 activity level significantly affects the processes in cell. Posttranslational stabilization of CYP2E1 seems to be the main mechanism of its regulation in living cell. It is known that degradation of CYP2El takes part in cytoplasmic proteasome system. The efficiency of such degradation depends on the presence of molecular chaperones (HSP90) as was shown from in vitro experiments. But the processes that involve HSP90 in the degradation of CYP2E1 and the mechanisms of transfer of microsomal CYP2E1 to the proteasome system remain unknown. This paper investigates HSP90-dependent processes in mechanisms of CYP2El degradation and the possible role of ethanol in them.

**Results:**

With the help of computational methods we have shown that CYP2E1 can interact with HSP90 resulting in dissociation of CYP2E1 from membrane and formation of the CYP2E1-HSP90 complex for its further transfer to the proteasome for degradation. The twofold increase of both CYP2E1 and HSP90 in the mouse liver under the constant alcohol administration was shown using WB methods. Also, as was shown in silico, ethanol molecule, while binding to the CYP2E1 active site, prevents its interaction with HSP90, thus resulting in accumulation of CYP2E1 in cell.

**Conclusions:**

Cytoplasmic HSP90 and membrane-bound CYP2E1 may directly interact with each other as partner proteins, leading to the dissociation of the CYP2E1 from the membrane. This makes it possible to transfer microsomal CYP2E1 in complex with HSP90 to the proteasome for proteolysis. The ethanol molecule inhibits the interaction of HSP90 with CYP2E1 leading to the suppression of its proteasome degradation, thus increasing level of this protein in the cell. Other substrates of CYP2E1 should increase level of this protein in the same way. This may be one of the mechanisms of substrate-dependent regulation of the CYP2E1 expression in the cell.

## Background

As the main role of microsomal CYP2E1 is detoxification of xenobiotics (exogenous low weight compounds) the highest level of its constitutive expression is found in liver and kidneys, organs that mostly utilize and excrete harmful substances off the organism [1,2]. There are 160 hydrophobic substances of exogenous origin known as substrates for CYP2E1 ( http://cpd.ibmh.msk.su/). Most of them are environmental pollutants (industrial wastes, fertilizers and solvents), components of food additives, drugs and cosmetics. CYP2E1 on one hand contributes in withdrawal of xenobiotics by metabolizing them in liver (thus taking active part in adaptation of organism to the adverse environmental factors). On the other hand CYP2E1, while metabolizing, may activate its substrates into cytotoxins, which causes different cell damages. Substrates of CYP2E1 induce its protein expression level but the mechanisms of such induction steel need to be investigated. Such increase in CYP2E1 protein level causes homeostasis misbalance in cell. Thus it is shown that introduction of ethanol (one of the most widely used CYP2E1 substrate) into the animals causes intensification of peroxidation processes and depletion of hepatocyte antioxidant system [3]. Activation of peroxidation processes may be caused by the ethanol-dependent induction of CYP2E1, which can generate oxygen radicals during its catalytic cycle. As a result an oxidation stress usually develops in cell [4]. Herewith ethanol does not only stimulate CYP2E1-dependent peroxidation, but serves as a source of free radicals itself (during the CYP2E1-dependent oxidation of ethanol a 1-hydroxyethyl radical is being formed [3,5]). Posttranslational stabilization of protein molecule with substrates is one of the main mechanisms of regulation of CYP2E1 expression level in cell. It is believed that substrates while in the active site of enzyme, change and stabilize protein structure, thus preventing its fast degradation with proteasomes [6-8]. The mechanisms of such substrates-dependent CYP2E1 protein stabilization mostly are staying uninvestigated. The processes of transfer of microsomal CYP2E1 to the proteasome (where its degradation takes place) also need to be investigated. It is thought that cytoplasm heat shock proteins, in particular HSP90, actively participate in these processes [9-11]. This work is devoted to investigating the role of HSP90 in the degradation of microsomal CYP2E1 and studying the influence of ethanol on these processes.

## Results and discussion

**Figure 1:**
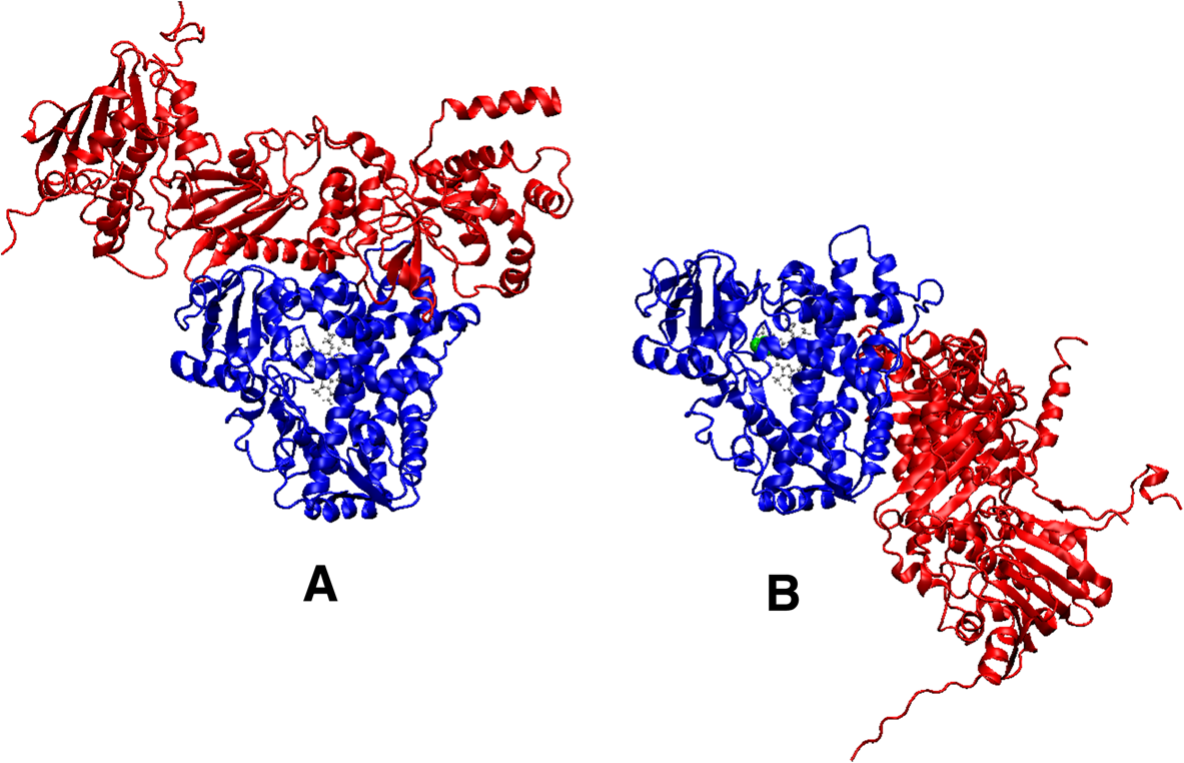
**Complexes of HSP90 with CYP2E1 (A) and CYP2E1-ethanol (B).** CYP2E1 is colored in blue and HSP90 – in red, ethanol molecule is shown as green spheres and heme molecule is shown as gray ball-and-stick.

**Figure 2:**
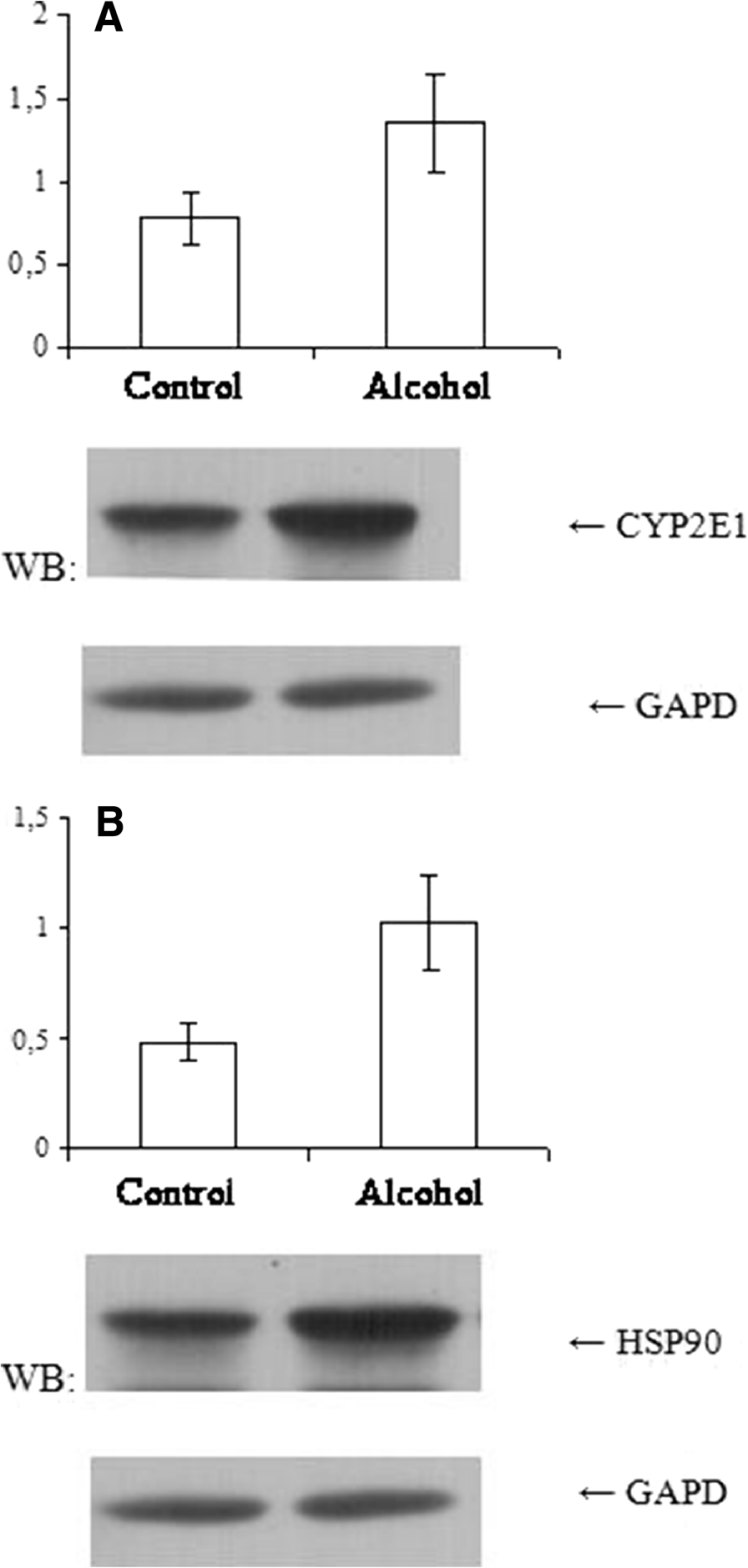
**CYP2E1 (A) and HSP90 (B) proteins expression in mouse liver under chronic action of ethanol.** Western blot analysis of total liver lysates probed by specific anti-CYP2E1 and anti-HSP90 antibodies is presented. GAPD is used as loading control.* P < 0,05 compared to control (n = 4–5).

It is known that the level of CYP2E1 expression in cell is regulated at all stages of protein biosynthesis from the initiation of transcription to the posttranslational stabilization of this enzyme [12]. Posttranslational substrate-dependent stabilization is one of the main mechanisms that regulates the intracellular content of CYP2E1 [12,13]. It is shown that the stabilization of CYP2E1 by the ethanol molecule increases its half-life period from 6 to 37 hours [7]. It is known that in the absence of substrate and with the participation of the enzymes of proteasome system the so-called rapid degradation of CYP2E1 is taking place. The in vitro experiments with heterologous recombinant proteins revealed that a cytoplasmic heat shock proteins could play a decisive role in the mechanisms of such degradation [11]. In particular, it was shown that specific inhibition of HSP90 resulted in the accumulation of microsomal CYP2E1 in cell [9,10]. However, HSP90-dependent processes of CYP2E1 degradation are poorly investigated today. It is also unclear how CYP2E1 is being transferred from membrane to the cytoplasm and becomes accessible to the proteasome degradation system. In order to investigate the HSP90-dependent processes in the CYP2E1 degradation mechanisms we have evaluated the possibility of direct spatial interactions of proteins by using computer simulation methods. During the protein-protein docking of CYP2E1 with HSP90 the CYP2E1-HSP90 complex was obtained. This complex (Figure 1A) is characterized in the Table 1. Due to this model, it was revealed that HSP90 binds to membrane-associated domain of CYP2E1 (the main external functionally active sites in the structure of CYP2E1 are presented in Table 2). Besides, two main structural and functional features of this complex could be distinguished from Tables 1 and 2. First of all, the entrance to the CYP2E1 active centre channel is being covered by HSP90 molecule, which reduces the probability of CYP2E1 interaction with substrates. This prevents protein structure stabilization by substrates and results in enzyme rapid degradation. Secondly, some hydrophobic region of CYP2E1 (primarily amino acid residues responsible for the association with the membrane) is also being screened by HSP90 molecule while forming CYP2E1-HSP90 complex. This promotes the microsomal CYP2E1 dissociation from the membrane and helps to shuttle it into the cytoplasm to the proteasome for proteolysis. The obtained results that allowed us to evaluate the studied proteins as partners confirmed data obtained by other authors during in vitro experiments with heterologous recombinant proteins [11]. It is known that substrate-dependent stabilization of the CYP2E1 molecule significantly inhibits its degradation [6]. It was shown that ethanol can structurally stabilize the CYP2E1 molecule and suppress proteolysis by the ethanol-dependent inhibition of the proteolytic system [8]. However, the mechanism of such an inhibition has not been fully understood yet. During our work the features of CYP2E1 and HSP90 expression under the chronic action of ethanol were studied. WB method revealed a twofold increase in CYP2E1 level in the liver of experimental mice, which consumed 10% ethanol in drinking water for 35 days (Figure 2A). Previously, no significant changes were shown in Cyp2e1 mRNA level relative to the control under similar experimental conditions [14]. The obtained data indicates that the increase in CYP2E1 protein level is caused by the stabilization of the enzyme molecule but not by the transcription process intensification. It is known that such substrate-dependent induction of CYP2E1 increases peroxidation processes and leads to the antioxidant system depletion and development of oxidative stress in the cell. This state is accompanied by intracellular structural and functional damages of macromolecules (such as denaturation, degradation, protein misfolding, etc.), thus in turn causing an intensification of the stress genes expression, in particular genes that encode proteins of the HSP family. The doubling of HSP90 content in the liver of experimental animals was found using WB method (Figure 2B). Such increase in intracellular stress proteins level is one of the main adaptive responses to oxidative stress and is aimed to restore the cell homeostasis [15]. The increase in the level of expression of the HSP90 should lead to the intensification of CYP2E1 proteolysis and reducing of its content in the cell. However, such effect was not observed in our experimental conditions. Therefore, we suggested that under the chronic effect of ethanol the interaction of the chaperone HSP90 with CYP2E1 is being inhibited thus blocking its HSP90-mediated transfer to the proteasome for subsequent degradation. In order to test this hypothesis we studied the effect of ethanol on the interactions of CYP2E1 with HSP90. For this purpose, a complex of CYP2E1 with ethanol (CYP2E1-ethanol) was designed and optimized using computer simulation methods. We have shown that ethanol molecule causes some decrease in volume and surface area of the CYP2E1 (Table 3). This may indicate the spatial stabilization of CYP2E1 molecule. Some changes in charges on protein surface and a significant decrease in the enzyme polar surface under the influence of ethanol were also shown (Table 3). The appearance of large positively charged region in the area of entrance to the active site and changes in the CYP2E1 protein surface charge and polar surface area can affect its ability to interact with other ligands and/or protein partners, HSP90 in particular. To study the effect of ethanol on the CYP2E1 interaction with HSP90, we performed a protein-protein docking of CYP2E1-ethanol with HSP90. The structure of CYP2E1-ethanol-HSP90 complex is presented at Figure 1B. The obtained data showed significant change in preferred HSP90 binding site of CYP2E1 (the detailed characteristics of CYP2E1-ethanol-HSP90 complex are presented in Methods section). Such difference in favourable binding sites may be explained by the changes in charged regions on protein surface in presence of ethanol. The obtained data shows that some structural adjustments in spatial structure of CYP2E1 under the influence of ethanol reduce the efficiency of its interaction with HSP90. Such ethanol-dependent inhibition of the CYP2E1-HSP90 complex formation leads to further inhibition of both the enzyme dissociation from the membrane and its subsequent degradation in the proteasome. This may explain the revealed elevation of CYP2E1 level in conjunction with a high level of HSP90 expression in the liver of animals chronically treated with ethanol. Based on these results we can assume that the cytoplasmic HSP90 and microsomal CYP2E1, as partner proteins, can directly interact with each other leading to the dissociation of the CYP2E1 from the membrane and its further transfer to the proteasome for proteolysis. The ethanol-dependent inhibition of the interaction between HSP90 and CYP2E1 suppresses its proteasome degradation, thus leading to its accumulation in the cell. The obtained data let us suggest that this is one of the mechanisms of substrate-dependent regulation of the CYP2E1 level in the cell.

**Table 1:** The main characteristics of HSP90 complexes with CYP2E1 and CYP2E1-ethanol

**Parameters**	**CYP2E1-HSP90**	**CYP2E1-ethanol-HSP90**
Binding energy (−ΔG_Gibbs_, kJ/mole)	800	470
Сontact area (Å2)	1941	1010
Resides of CYP2E1 involved in contact with HSP90	L32, F37, P38, P40, I41, Y71, S74, Q75, R76, D102, P104, H107, A108, H109, R110, D111, R112, G119, P120, T121, R198, Y218, P222, S223, L225, H226, I236, H232, R233, K237, A240, E241, K243, E244, Y245, S247, E248, K251, A280, E281, M286, D287, T290, V291, R374, D375, L382, K385, G386 (49)	N135, Y136, G139, K140, Q141, G142, E144, S145, Q148, R149, H152, F153, E156, R159, K160, Q162, K187, S336, R337, I338, A340, P491 (22)

**Table 2:** The main domains and functionally active sites in the structure of CYP2E1

	**Localization (residue number from N-term)**
Membrane-associated domain	1-96, 368-390
Cytoplasmic domain	97-367, 391-491
Active site and its channel	41-57, 70–79, 113–119, 202–218, 298–305, 387–396, 467-471

**Table 3:** Comparative characteristics of the CYP2E1 and CYP2E1-ethanol spatial structures

	**CYP2E1**	**CYP2E1-ethanol**
Volume (Å3)	41147.4	39364.8245
Gyration radius (Å)	22,574	22.671
Polar surface area (Å2)	16936,1	15985.5462
Surface area (Å2)	54400.7500	53120.5892
RMSD – Cα (Å)	–	0.718
RMSD – all atoms (Å)	–	1.9

## Conclusions

Cytoplasmic chaperone HSP90 and membrane-bound CYP2E1, as partner proteins may directly interact with each other, leading to the dissociation of the CYP2E1 from the membrane. This makes it possible to transfer microsomal CYP2E1 to the proteasome for proteolysis. The ethanol molecule inhibits the interaction of HSP90 with CYP2E1 leading to the increased content of this protein in the cell. We assume that other substrates of CYP2E1 should increase its content in the same way. This may be one of the mechanisms of substrate-dependent regulation of the CYP2E1 level in the cell.

## Methods

### Animals

The Institute of Molecular Biology and Genetics Bioethics Committee (head Prof. Dr. Lukash LL) according to the "Recommendations of the ethics committees that carry out the examination of biomedical research" (WHO, 2000), the order of the AMS of Ukraine № 50 from 06.07.2001 "On establishment of committees of medical ethics in research institutions of Academy of Sciences of Ukraine" and the "General ethical principles of animal experiments" (1 National Congress on Bioethics, Kyiv, 2001) approved procedures involved in the breeding and handling of animals (protocol № 10 dated 24.09.2008).

We used BALB/c mouse males, 3.5 months old, with the average weight of 30 g from vivarium of the Institute of Molecular Biology and Genetics of the National Academy of Sciences of Ukraine (Kyiv) in our work. Mice were kept at standard conditions with inverted diurnal light regime (8 night hours), at temperature 18-20°C and on standard diet. Animals were divided into two groups: experimental and control (intact). The experimental mice were fed with 10% ethanol in water. On day 35 mice of both groups were decapitated under the light ether anaesthesia.

### Western blotting and protein measurement

The relative level of CYP2E1 and HSP90 protein in the liver was determined by Western blot analysis. The liver tissues were homogenized in ice-cold RIPA-buffer (1:3) containing 20 mM TrisHCl, pH 7.5; 0.15 M NaCl; 1 mM EDTA; 1% NP-40; 1% sodium deoxycholate; 0.1% SDS and 1 mM protease inhibitor PMSF was added to liver tissue frozen in liquid nitrogen. The extraction of proteins was carried out on ice for 45 minutes. Centrifugation was then carried out at 11 000 g. for 20 min. at + 4°C. The protein concentration in the supernatant was determined [16]. Proteins from the liver of each mouse (50 μg per line) were separated using 12% polyacrylamide gel with 0.1% SDS [17]. The semi-dry electrotransfer of proteins to the nitrocellulose membranes was held at 200 mA for 40 minutes. Western blot analysis was held in the following way: nitrocellulose membranes were pre-incubated in 4% nonfat milk (Sigma, USA) in PBST-buffer, and then treated with polyclonal anti-CYP2E1 or anti-HSP90 antibodies (obtained in Rabbit in our institute earlier) diluted in 4% nonfat milk for 1 hour at room temperature. Membranes were incubated with peroxidase-conjugated secondary anti-Rabbit antibodies (Sigma, USA) for 1 hour. The GAPD (used as control) was identified using anti-GAPD antibodies (obtained in Rabbit in our institute earlier). The treatment of membranes with secondary antibodies was followed by chemiluminescence detection according to manufacturers’ instructions (Pierce). Membranes were exposed to autoradiography film (Agfa, Belgium) for 0,5 to 1 minutes. Digital images of immunoblots were analyzed using densitometric scanning analysis program Scion image 3.53.346.0 ( http://www.scioncorp.com/). The level of CYP2E1 and HSP90 protein was calculated as the ratio of protein values to GAPD and presented as relative units.

### Statistical analysis

Statistical analysis was performed using STATISTICA 7.0 (StatSoft, Inc. 2004, USA). Results are presented as mean ± standard deviation (SD). Differences between groups were identified using an unpaired two-tailed distribution of Student’s T test. P values < 0.05 were considered to be statistically significant.

### Computational work

**Figure 3:**
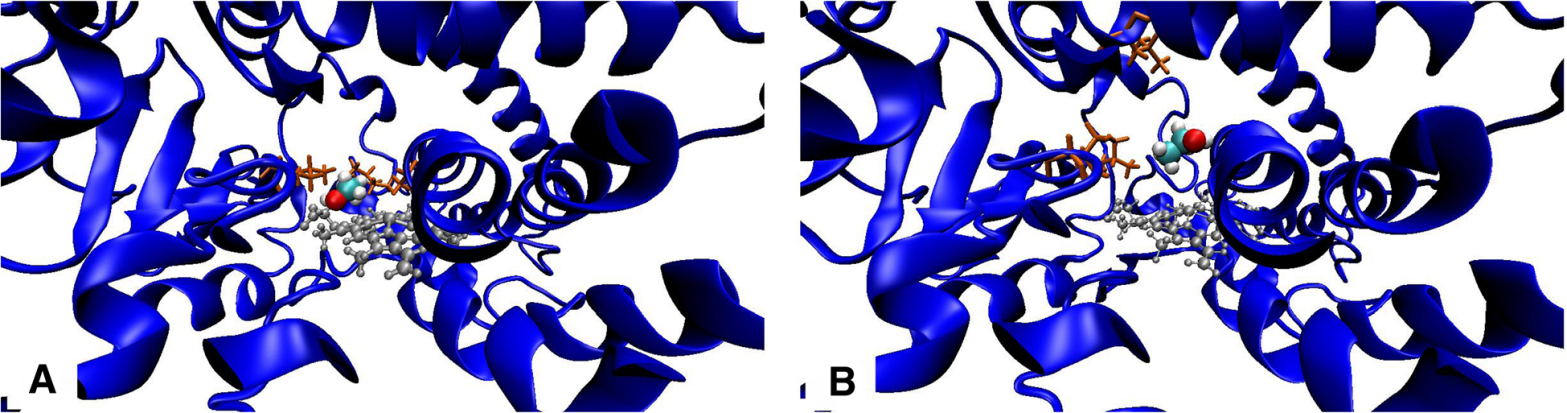
**Changes in the amino acid environment of ethanol molecule in the CYP2E1 active center during MD optimization. A** – before and **B** – after MD optimization. Residues that participate in the orientation of the ligand are shown as orange sticks; heme is shown as gray sticks and ethanol molecule – as spheres.

**Figure 4:**
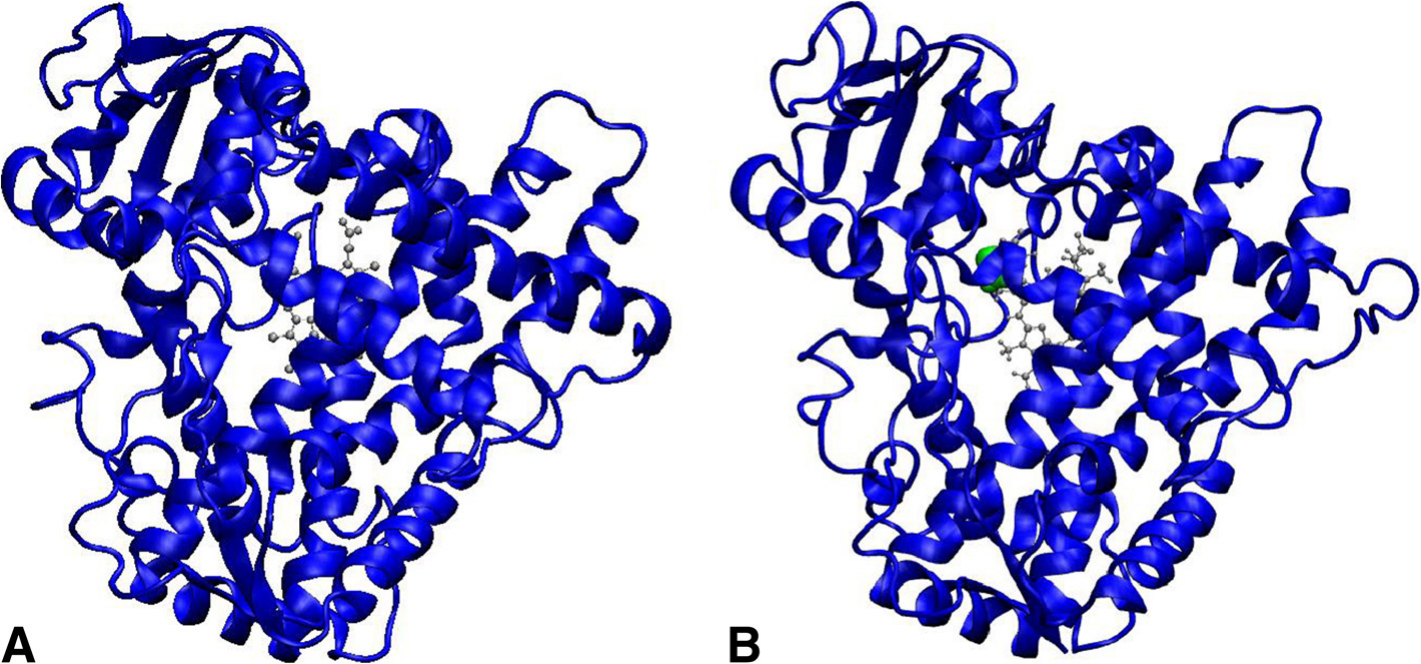
**The comparison of spatial structures of CYP2E1 (A) and CYP2E1-ethanol (B).** Heme is show as grey ball-and-stick and ethanol – as green spheres.

**Figure 5:**
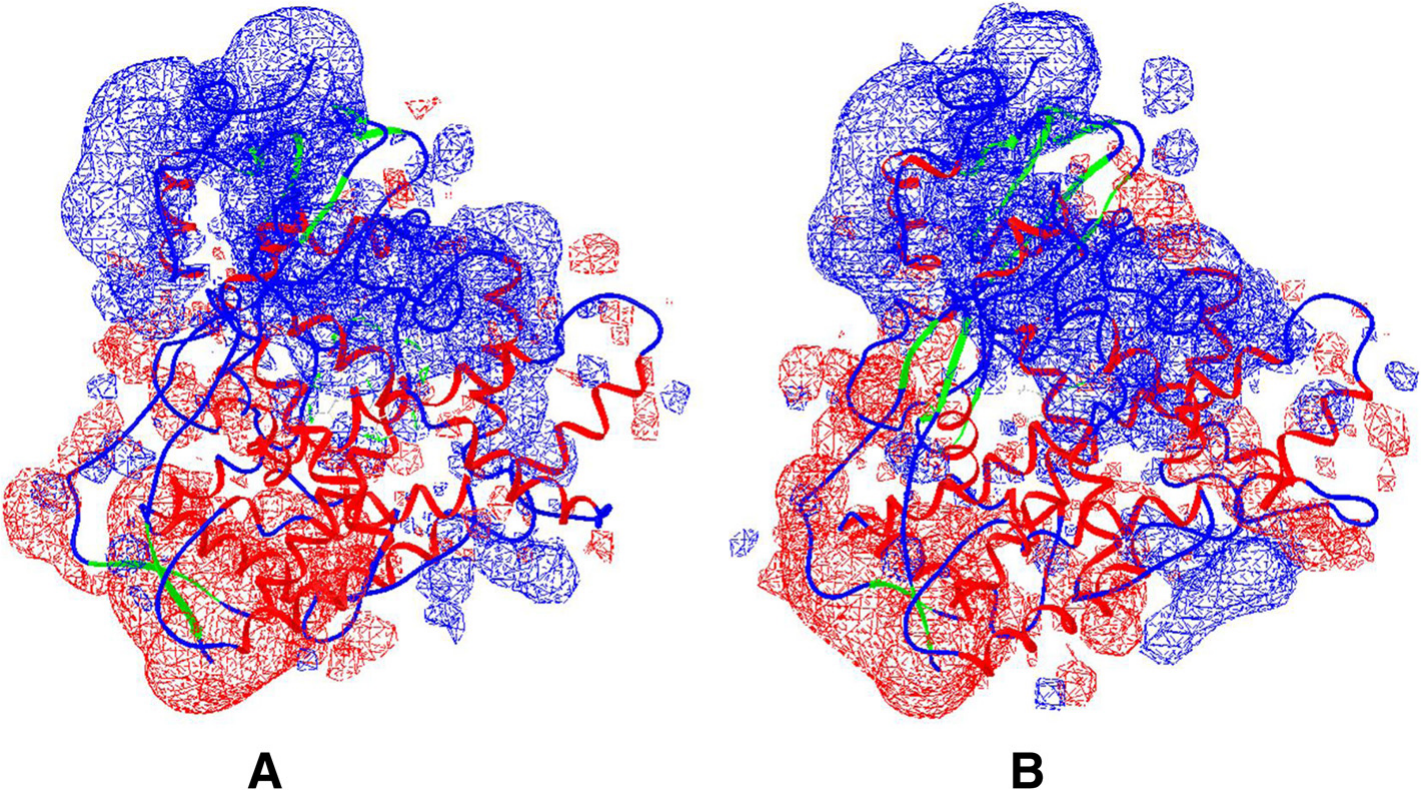
**The charged surface of the CYP2E1 (A) and CYP2E1-ethanol (B).** Blue shows negatively and red – positively charged protein regions.

Similar to the experimental data [11] we used computational model of recombinant CYP2E1 with deleted 30 a.a. at the N-terminal (membrane anchor) previously created and optimized by us [18]. The computational model of human HSP90 was built using SWISS-MODEL web-server ( http://swissmodel.expasy.org/). Protein-protein docking of CYP2E1 with HSP90 was made using HEX6.1-CUDA program with 5D FFT rotational correlation mode based on shape and electrostatics with subsequent molecular energy minimization for post processing. During this docking several CYP2E1-HSP90 complexes were obtained, whereof the most energetically favourable one (maximum energy of interaction -ΔG_Gibbs_ = 800kJ/mol) was selected. A complex of CYP2E1 with ethanol (CYP2E1-ethanol) was created and optimized using MD to investigate the influence of ethanol molecule on protein spatial structure. The docking of ethanol into the protein active site was performed using ArgusLab 4.01 program. The site of interaction with this ligand is located in the active site of the enzyme at a distance of 0,35-0,45 nm from the atom of heme iron and is characterized by high interaction energy (ΔG_Gibbs_=−19.72 kJ/mol) and a large contact area with CYP2E1. MD simulation of the CYP2E1-ethanol molecule was performed using NAMD soft [19]. It was solvated with TIP3P water using VMD soft [20]. We used standard conditions of NVE-ensemble at the temperature of 310K (37°C). Simulation was conducted for 1 ns. During MD optimization some changes were shown in the ethanol molecule orientation inside the enzyme active site (amino acid environment of the ethanol molecule changed from Leu115, Ala299, Leu368 and heme to Leu210, Val364, Leu368 and heme, see Figure 3), causing minor protein structure rearrangements (Figure 4). Some characteristics of CYP2E1 spatial structure and their changes caused by ethanol are presented in Table 3. Besides, the ethanol molecule caused changes in charges on CYP2E1 surface (Figure 5). Thus large positively charged region appears in the area of entrance to the active site channel. A negatively charged area has significantly increased in the region of CYP2E1 that corresponds to the cytoplasmic domain. Protein-protein docking of the CYP2E1-ethanol with HSP90 was the same as for the complex of CYP2E1 with HSP90. The cytoplasmic domain of CYP2E1 showed the highest energy of binding with HSP90. The enzyme membrane-bound domain showed significantly lower energy of binding with HSP90 (data not shown). Also a significant reduction was shown in the interaction area and in the number of residues involved in the contact (Table 1).

## Competing interests

The authors declare that they have no competing interests

## Authors’ contributions

KVO carried out the computational work and drafted the manuscript. MOV worked with experimental animals, carried out the immunoassays, participated in the design of the study and performed the statistical analysis. CMO conceived of the study, and participated in its design and coordination and helped to draft the manuscript. All authors read and approved the final manuscript.
